# Hospital Meetings

**Published:** 1903-08-01

**Authors:** 


### HOSPITAL MEETINGS.

### QUEEN CHARLOTTE'S LYING-IN HOSPITAL.

The annual general meeting of this hospital was held at 22 Portman Square on July 23rd. The chair was taken by Viscount Portman, President of the hospital, who read a letter from the Earl of Hardwicke expressing regret at his inability to attend, and quoting figures illustrative of the progress in the work of the hospital. In 1882, 692 patients passed through the hospital; in 1892 the number was 973, and in 1902 it had risen to 1,271. For the first six months of this year it was 722. The chairman expressed his firm conviction that there was no charitable institution in London more deserving of support than Queen Charlotte's Hospital, one of its greatest claims being on account of the large number of infant lives saved by its means.

The Bishop of Kensington moved the following resolution: "That this meeting of Governors and friends of Queen Charlotte's Hospital desires to express its continued interest in the charity, and having regard to its great and growing work, to commend to public support the efforts now being made by the committee to provide the further increased accommodation necessary for patients and nurses, and to obtain the additional funds needed for maintenance." The Bishop said that Queen Charlotte's Hospital was situated in that part of London for which he was officially responsible; he went over it recently and was very much struck not only by the completeness and thoroughness of the arrangements of the hospital, but also by the tenderness and care shown towards the patients. There were more than twenty hospitals situated in his district and he had been over all of them but nowhere were the qualities above-mentioned so prominent as in Queen Charlotte's hospital. Referring to the efforts of the committee to provide further accommodation for patients and nurses, the Bishop recalled the time when the comforts and convenience of the nursing staff were very little attended to, but of recent years a great improvement had taken place in this respect. He had been over the nurses' quarters at this hospital and thought that every care was taken to render them as comfortable as possible. The statistics that appeared in the report were really marvellous; with 1,271 in-patients during the year the number of deaths, only five, was most remarkable; over 1,200 out-patients had also been treated and these received a very large amount of attention.

The resolution was seconded by Lieut.-Gen. Lowrie.

Mr. H. A. Harben (vice-chairman) moved the second resolution: "That the work in which the Midwifery Training School has been engaged for many years past, in training medical pupils, midwives for the poor, and monthly nurses, is one of great importance in the interests of all classes of the community, and constitutes a high claim to the support of the public." Mr. Harben pointed out that almost every hospital of importance had now a medical school connected with it, as it was now beginning to be recognised that a first-class hospital could not exist in a satisfactory condition without a good medical school, and the converse was equally true. This hospital had always made the training of nurses and midwives special objects of attention. After alluding to the Act for the Registration of Midwives, Mr. Harben said he hoped that the restrictions that were to be laid down by the Central Board of Midwives would not be too strict. The Queen Charlotte's Hospital had taken steps towards improving the training of midwives, and had held a conference of all the lying-in hospitals in London to consider whether the training was sufficient. The majority of the pupils now underwent the full course of four or five months' training. Should the Central Midwives Board insist on a higher training, the hospital would give it. It had been suggested that a new training school for midwives should be established which should in a great measure supplant the Queen Charlotte's Hospital. He fully admitted that in many parts of London more hospitals were needed, and that no doubt the lying-in hospitals might be extended, but the assumption of those who advocated the establishment of a new training school for midwives was that there was no existing institution capable of performing that work. But he was prepared to maintain that the Queen Charlotte's Hospital was capable of carrying out any work of the kind; it possessed great experience and an admirable organisation and was capable of indefinite expansion. He could not help thinking that it would be a public misfortune if subscriptions were diverted from the Queen Charlotte's Hospital, and were devoted to other objects however worthy. He appealed most earnestly to the public not to cripple existing institutions.

The resolution was seconded by Mr. W. Rivers Pollock, who said he did not see how more training could be fitted in with the amount of time at the disposal of the pupils, who were all naturally anxious to begin to earn their livings. A great many students came to learn this special branch of medical training, because there was no place in London where they could be taught in the same way to deal with very small children.

A vote of thanks to Viscount Portman for presiding was moved by Colonel H. B. Hamilton, and seconded by the Rev. Vincent Borradaile.

The total ordinary expenditure for 1902 was £5,283, and the income £3,771. The legacies received amounted to £1,808, a portion of which was invested, and the remainder was used for the current expenses for the year. Increased grants were received from all the three great hospital funds. It has been resolved to enlarge the nurses' home by adding a story, so as to provide additional room for the nurses. A sum of upwards of £2,000 is required for this purpose.

### NATIONAL ORTHOPÆDIC HOSPITAL.

AMALGAMATION WITH THE ROYAL COMPLETED.

A SPECIAL general meeting of the governors and subscribers of the National Orthopædic Hospital was held on Friday, July 24th, to pass a resolution approving of amalgamation with the Royal Orthopædic Hospital, Mr. Robert Cooper, chairman of the committee, presiding.

The Chairman said no doubt there were those present who remembered the small lines on which that institution was commenced 25 years ago and the quiet way it did its work of usefulness among the 25 to 30 patients, the outside limit they were then able to accommodate. During the whole of the time the committee had been working in complete harmony with the staff. The work had steadily progressed; the hospital had been enlarged, but notwithstanding this they found themselves unable to cope with the claims daily coming before them. The committee therefore came to the conclusion that they must make a violent effort with a view to further extension. This came to the knowledge of the King's Fund, who, recognising the usefulness of the institution, gave them £500, and expressed a desire that it might be substantially enlarged so as to cope with all the wants of London. This was followed by the suggestion that they might amalgamate with the Royal Orthopædic and so by a single code and management save considerably in expenses. This fell in with the views of the committee and for some time negotiations had been going on. The conditions for the amalgamation had been laid down and it only remained for that meeting to say whether or no it should take place, leaving the details to the committee to arrange. The intention was to enlarge the site towards Regent's Park—in which they had been most generously assisted by Lord Howard de Walden—and that the new hospital—to be called the Orthopædic Hospital—should spring up on the site of their present buildings with the addition mentioned. Their identity would thus remain; the position was convenient for patients, being close to the railway termini and to Portland Road station; they were close to the surgeons, and also to Baker Street station, with which their projected outpost would be connected. They proposed to build a convalescent home in the country to accommodate 100 beds, within ten or twelve miles of town, so that the doctors would be able to reach the patients. They were in entire agreement with their staff in thi matter, and he would read a letter addressed to the committe by the staff as showing the keen interest they were taking in the subject. The letter was as follows:—

*To the Chairman of the Committee of Management.*

July 22nd, 1903.

DEAR SIR,—At a meeting of the Medical Board held at the hospital this afternoon I was desired to ask you to lay before the Governors at their special general meeting on the 24th inst. the following resolution which was unanimously passed by the Board:—

"That it having been reported in the press that on the amalgamation of the 'Royal' and the 'National' Orthopædic Hospital the surgical staffs of the two hospitals, as at present constituted, would be joined together, the medical staff of this hospital desire emphatically to state, at the special general meeting of Governors called for July 24th to consider the question of amalgamation, their absolutely unanimous opinion that all members of the surgical staff of the amalgamated hospitals—without any exception—must be 'F.R.C.S.' England, which stipulation they have understood from the Chairman of the Committee of Management has always been put forward by him in the negotiations with the King's Hospital Fund."

I remain, dear sir,
Yours very truly,
(Signed) E. MUIRHEAD LITTLE,
Hon. Secretary, Medical Board.

The Chairman concluded by moving a resolution that the amalgamation be carried out. Mr. Hoblyn, the Vice-Chairman, seconded it, and it was carried *nem con.*

Mr. Little, F.R.C.S., one of the surgeons to the hospital, said the reason for the resolution adopted by the medical board, which had been read to the meeting, was that they wished the new hospital to have the rule which had always been maintained in the National, which applied to all the general hospitals of London and to most of the special hospitals of repute, but which, unfortunately, had not been strictly adhered to in the Royal, or the City Orthopædic. They wanted it made clear so that it should not be possible afterwards for anyone to say there had been any misapprehension on the subject.

Mr. Tubby, F.R C.S., another member of the surgical staff, emphasised the point. They felt, he said, that the surgeons should have as high a qualification as possible.

The Chairman, in declaring the proceedings at an end, said that after that meeting they would be able to offer their neighbours the right hand of fellowship, and he hoped and believed the usefulness of both would be enhanced. He would like to say that they had taken great care to preserve their Ladies' Committee intact to carry on the beneficent work that had been the admiration of them all.

